# HMGB1 and Histones Play a Significant Role in Inducing Systemic Inflammation and Multiple Organ Dysfunctions in Severe Acute Pancreatitis

**DOI:** 10.1155/2017/1817564

**Published:** 2017-02-21

**Authors:** Runkuan Yang, Jyrki Tenhunen, Tor Inge Tonnessen

**Affiliations:** ^1^Department of Intensive Care Medicine, Tampere University Hospital, University of Tampere, 10 Biokatu, 33014 Tampere, Finland; ^2^Department of Critical Care Medicine, University of Pittsburgh Medical School, 3550 Terrace Street, Pittsburgh, PA 15261, USA; ^3^Department of Emergencies and Critical Care, Oslo University Hospital, 4950 Nydalen, 0424 Oslo, Norway; ^4^Department of Surgical Science, Anesthesiology and Intensive Care Medicine, Uppsala University, 751 85 Uppsala, Sweden; ^5^Institute of Clinical Medicine, University of Oslo, Blindern, 0316 Oslo, Norway

## Abstract

Severe acute pancreatitis (SAP) starts as a local inflammation of pancreatic tissue that induces the development of multiple extrapancreatic organs dysfunction; however, the underlying mechanisms are still not clear. Ischemia-reperfusion, circulating inflammatory cytokines, and possible bile cytokines significantly contribute to gut mucosal injury and intestinal bacterial translocation (BT) during SAP. Circulating HMGB1 level is significantly increased in SAP patients and HMGB1 is an important factor that mediates (at least partly) gut BT during SAP. Gut BT plays a critical role in triggering/inducing systemic inflammation/sepsis in critical illness, and profound systemic inflammatory response syndrome (SIRS) can lead to multiple organ dysfunction syndrome (MODS) during SAP, and systemic inflammation with multiorgan dysfunction is the cause of death in experimental SAP. Therefore, HMGB1 is an important factor that links gut BT and systemic inflammation. Furthermore, HMGB1 significantly contributes to multiple organ injuries. The SAP patients also have significantly increased circulating histones and cell-free DNAs levels, which can reflect the disease severity and contribute to multiple organ injuries in SAP. Hepatic Kupffer cells (KCs) are the predominant source of circulating inflammatory cytokines in SAP, and new evidence indicates that hepatocyte is another important source of circulating HMGB1 in SAP; therefore, treating the liver injury is important in SAP.

## 1. Introduction

Acute pancreatitis (AP) is a relatively common disease, its severe form is potentially fatal, and SAP is associated with high mortality, ranging from 15 to 40% [1–8]. AP starts as a local inflammation of pancreatic tissue that induces the development of multiple extrapancreatic organs dysfunction including acute lung injury [8–10], general endothelial barrier dysfunction, liver injury, and gut barrier dysfunction [2, 6, 8]; systemic inflammation is thought to be the key link to multiple organ dysfunction (MOD) in SAP [11, 12] and gut BT plays a critical role in triggering/inducing systemic inflammation [13, 14]; however, the underlying mechanism of BT in SAP is still poorly understood. The inflammatory cytokines play a crucial role in the pathogenesis of SAP [6, 7, 15, 16] and the late proinflammatory cytokine HMGB1 is particularly important in SAP [17, 18], because the circulating HMGB1 levels can reflect the disease severity and are potent in augmenting systemic inflammation [17, 18], and emerging evidence shows that HMGB1 is also an important factor that mediates 85% of the gut BT in acetaminophen hepatotoxicity [19]; therefore, it is possible that HMGB1 also mediates BT in SAP. Except HMGB1, new investigations show that extracellular histones and DNAs might also significantly contribute to multiple organ injury during SAP [20–23]. Because hepatic KCs are the predominant source of circulating inflammatory cytokines (including HMGB1) in SAP [8], and new evidence indicates that hepatocyte is another important source of circulating HMGB1 in SAP [15, 16]; therefore, the inflamed liver is an important contributor to the circulating HMGB1 and the liver is a promising therapeutic target to prevent BT and systemic inflammation during SAP. This manuscript focuses on the new development in gut BT and provides evidences to show that HMGB1, histones, and DNAs might significantly contribute to systemic inflammation and multiple organ injury during SAP.

### 1.1. Ischemia-Reperfusion, Circulating Inflammatory Cytokines, and Possible Bile Cytokines Contribute to Gut Barrier Dysfunction in SAP

Gut barrier dysfunction is frequently seen in SAP [6, 11, 24, 25] and clarifying the underlying mechanisms is important because the intestine is the biggest reservoir of bacteria in the body and leakage of bacteria or microbial products, notably LPS, from the intestinal lumen into the systemic circulation, leads to initiation or amplification of a systemic inflammation and MOD [26].

Intestinal ischemia-reperfusion significantly contributes to small intestinal injury during SAP [27, 28] by inducing gut mucosal injury and affecting gut muscular layer to impair gut motility, which is frequently seen in SAP [14], and the reduced intestinal motility significantly contributes to disrupted intestinal microflora and BT [14].

The circulating inflammatory cytokines including TNF-*α*, IL-6, and HMGB1 also contribute to gut mucosal injury [29–32], among these inflammatory cytokines, extracellular HMGB1 triggers and sustains the inflammatory response by inducing cytokine release and by recruiting leucocytes [33]. However, HMGB1 undergoes extensive posttranslational modifications, in particular acetylation and oxidation, which significantly modulate the functions of HMGB1 [33, 34]. High levels of serum HMGB1, in particular of the hyperacetylated and disulfide isoforms, are sensitive disease biomarkers [33], and SAP patients have significantly increased serum HMGB1 levels [35, 36], which can reflect the severity of gut barrier injury [35]. The circulating HMGB1 contributes to gut mucosal hyperpermeability and induces evident BT during hemorrhagic shock and reperfusion [37], and exogenous HMGB1 injection is able to induce gut hyperpermeability and BT in normal mice [29].

Except from the circulating HMGB1, bile HMGB1 might also significantly contribute to intestinal mucosa injury and induces evident BT in SAP because SAP patients have significantly increased circulating LPS levels [38], and LPS injection reduces 40% of bile flow in normal rats [31]; adequate bile is important to maintain gut epithelial tight junction and intestinal bacterial homeostasis [31] and decreased gut luminal bile volume not only impairs intestinal tight junction, but also changes intestinal bacterial homeostasis to facilitate BT [31]. In addition, LPS injection (to normal animals) markedly increases bile TNF-*α* and HMGB1 levels, and the endotoxemic bile can induce gut mucosal hyperpermeability and evident BT in normal mice [31], and neutralization of bile HMGB1 can reverse endotoxemic bile induced intestinal mucosal hyperpermeability and BT in normal mice [31], suggesting that bile HMGB1 is able to cause gut mucosal injury and intestinal BT.

### 1.2. HMGB1 Plays a Significant Role in Mediating BT in SAP

SAP induces significant small intestinal injury [27, 28], and the failure of gut barrier is associated with BT [27], which is evident in SAP [6, 24, 25]. SAP patients have significantly increased serum LPS level, which is likely derived from the gut lumen or from translocated intestinal bacteria [38]. 68.8% of the SAP patients have bacteraemia and these bacteria have been tested as gut-derived opportunistic pathogens [24], which likely derive from small bowel rather than from the colon [25]. Therefore, it is important to identify the key factor that mediates intestinal BT in SAP. Gut bacteria can adhere to the injured intestinal mucosa that is necessary but not sufficient to induce gut BT in which HMGB1 and other unknown factors are also needed, because evidence shows that exogenous HMGB1 injection can induce gut mucosal injury and evident BT in normal mice [29], and HMGB1 also contributes to gut barrier dysfunction in rats with SAP [32]; blockade of HMGB1 reduces 85% of BT but does not significantly improve gut mucosal injury during acetaminophen hepatotoxicity [19]; similarly, ethyl pyruvate (EP), which is a HMGB1 inhibitor [39], reduces 80% of the gut BT but did not significantly decrease intestinal mucosal hyperpermeability in a lethal SAP animal model [6]; this BT-inhibition effect is associated with EP inhibiting 80% of the hepatic tissue HMGB1 release [15]. These evidences indicate that HMGB1 might mediate gut BT in SAP, and this hypothesis is confirmed by the following experiment: neutralization of HMGB1 decreased 70% of the gut BT (4252 ± 205 CFU/g for the sham antibody group versus 1235 ± 41 CFU/g for the anti-HMGB1 group, results are mean ± SEM, *n* = 6 for each group, unpublished data) but did not reduce gut mucosal hyperpermeability in another commonly used murine SAP model (7 hourly 50 *μ*g/kg intraperitoneal injections of cerulean and a single intraperitoneal injection of 4 mg/kg* Escherichia coli* lipopolysaccharide). These data indicate that intestinal mucosal injury is essential but not sufficient for gut BT during SAP; HMGB1 plays a significant role in mediating (at least partly) gut BT in experimental SAP and BT is likely an active “transcellular” procedure.

### 1.3. Gut BT Induces/Triggers Systemic Inflammation and Sepsis

Gut BT not only contributes to pancreatic infection [14, 25], but also induces/triggers systemic inflammation/sepsis in critical illness [13, 14], and the profound systemic inflammation can lead to MOD and mortality in SAP [9, 14, 27, 40]. This is one of the important underlying mechanisms that SAP frequently affects extrapancreatic organs [3, 6], and the incidence of MOD in SAP is not available but certainly higher than the 20–30% of mortality rate in SAP [3]. Systemic inflammation with multiorgan dysfunction is the cause of death in a murine ligation-induced SAP, and systemic inflammation and MOD can lead to the preponderance of mortality (75%) in this lethal SAP model [11] while bile duct ligation does not have mortality [11]. Therefore, the gut is thought to act as the starter of SIRS [27] and HMGB1 is an important factor that links gut barrier dysfunction and MOD during SAP.

### 1.4. The Liver Itself Plays an Important Role in BT in SAP

AP frequently affects the liver [6, 15] that plays an important role in BT during SAP by virtue of its unique structure and immune surveillance function [14, 41]. The liver is the largest organ in the body, hepatocytes account for 70–80% of the hepatic cytoplasmic mass and nonparenchymal cells make up 20–30% of the hepatic cytoplasmic mass [41]. Among the nonparenchymal cells, KCs, sinusoidal endothelial cells, and the natural killer (NK) lymphocytes exert cellular defence functions for the whole body but also for the liver itself [41]. The liver has a large number of immune cells that can be rapidly expanded in response to infection or injury by recruiting leukocytes from the circulations [42]. SAP frequently injures the liver [6, 15, 16, 40] and impairs the phagocytic function [14, 40]; the impaired host defence fails to clear regional lymph nodes and resultantly facilitates BT [14, 40].

### 1.5. HMGB1, Extracellular Histones, and DNAs Contribute to Multiple Organ Injuries

About 20–30% of acute pancreatitis patients develop SAP in clinical practice; the mortality rate in SAP is 20–30% [3]; however, most of the SAP related mortality is not due to pancreatic injury itself; systemic inflammation with multiorgan dysfunction is the cause of death [11]. Early inflammatory cytokines are certainly involved in the pathogenesis of SAP; however, due to the narrow window time of the early cytokines in clinic, the clinical significance of the early inflammatory cytokines is limited. Attention should be focused on the late inflammatory cytokines such as HMGB1 and the newly recognized histones because these damage associated molecular patterns (DAMPs) have much wider window time to treat the patients and these DAMPs also significantly contribute to multiple organ injuries. The circulating HMGB1 level is significantly higher in SAP patients than in patients with mild pancreatitis [43]; the serum HMGB1 levels are higher in SAP patients with organ dysfunction and infection than in patients without organ dysfunction or infection [36]; the serum HMGB1 levels in nonsurvivors are higher than those in survivors [36]; serum HMGB1 levels are positively correlated with disease severity scores [36]. Pancreatic tissue HMGB1 levels are significantly increased in experimental SAP [44]; HMGB1 promotes the pathogenesis of pancreatitis [39, 45], and inhibiting HMGB1 therapy ameliorates the pancreatic injury [46]. HMGB1 contributes to liver injury in ischemia-reperfusion [47]. Exogenous HMGB1 injection is able to induce liver injury in normal mice [29]. HMGB1 impairs hepatocyte regeneration during acetaminophen hepatotoxicity and blockade of HMGB1 improves hepatocyte regeneration in acetaminophen overdose-induced fatal liver injury [48]. Anti-HMGB1 treatment protects against APAP hepatotoxicity in rats [49]. HMGB1 also contributes to renal ischemia-reperfusion injury [50], sepsis-induced kidney injury [51], and severe acute pancreatitis related kidney injury [52]. HMGB1 is also found to significantly contribute to hemorrhagic shock related acute lung injury (ALI) [53], hypoxia-induced ALI [54], and severe acute pancreatitis related ALI [55].

The extracellular histones are another group of important DAMPs molecules [56], which are toxic to host cells and elicit immunostimulatory effect that results in tissue damage and inflammation [56, 57]. The necrotic tissue/the dying cells release HMGB1 and histones as DAMPs [57]; therefore, circulating histones are significantly increased in both SAP patients and experimental animals with SAP [20–23] and the circulating histones levels reflect the disease severity in experimental AP [21].

The on-admission circulating nucleosome (containing DNA and histones) levels can predict organ dysfunction during the hospitalization of AP patients [20], suggesting that extracellular histones might contribute to multiple organ dysfunctions in SAP. Reduced pancreatic injury is associated with decreased histones 3 and histone 4 levels in the pancreas in response to taurocholate challenge [23], suggesting that histones are associated with pancreatic injury during SAP. The extracellular histones kill endothelial cells and are one of the major mediators of death in sepsis [58]. Extracellular histones contribute to two different acute fatal liver injury models [57]; TLR2 and TLR4 are the major receptors for extracellular histone mediated sterile inflammation, tissue injury, and organ failure in acute liver failure (ALF) [57]; neutralization of histones can ameliorate these two different fatal liver injury models in mice [57]. Extracellular histones can induce microvascular endothelial injury and the TLR2/4-mediated inflammation can lead to acute tubular necrosis in acute kidney injury (AKI) [59, 60]. Extracellular histones injure endothelial cells causing microvascular thrombosis and hemorrhage in acute lung injury (ALI) [61]. Histones also contribute to stroke in acute brain injury, and blockade of histones can reduce infarct size [62]. Histone H4 and increased circulating neutrophil extracellular traps (NETs) activate platelets, which trigger platelet aggregation and clotting that result in microvascular thrombosis and vascular and parenchymal injury in sepsis [57, 58, 60]. Therefore, extracellular histones contribute to the microvascular complications of sepsis, small vessel vasculitis, acute fatal liver injury, acute kidney, and acute lung injuries [58, 60].

Cell-free plasma DNA is significantly increased in SAP patients [20, 22] and in experimental animal with SAP [22]. Damaged cells from SAP can release nucleosomes, which contain DNA and histones, into the circulation to promote inflammation, and the circulating nucleosomes levels can predict AP patients who present no clinical signs of organ dysfunction on admission and are bound to develop organ dysfunction during hospitalization [20], suggesting that DNA/or histones likely play a significant role in the development of organ dysfunction during SAP. Neutrophils play an active role in the development of AP [22]. Except the secretion of antimicrobial compounds, activated neutrophils eliminate invading microorganisms by expelling nuclear DNA and histones to form extracellular web-like structures called neutrophil extracellular traps (NETs) [22]. NETs form in the pancreas in a murine SAP model; addition of NETs and histones to the acinar cells induces trypsin formation and activates the signal transducer and transcription [22]. NETs are able to activate platelets leading to thrombosis and the major contributor to this process is histone 4 [52]. NETs contribute to organ dysfunction in patients with infectious diseases and regulate organ inflammation and injury in mice with AP [22]. SAP patients have increased plasma levels of NETs [22]. Administration of DNase 1 (to deplete DNA and NETs) decreases circulating HMGB1 level and reduces neutrophil infiltration and tissue damage in the inflamed pancreas and lung [22], suggesting that DNA also significantly contributes to the development of SAP.

### 1.6. The Inflamed Liver Is an Important Source of Circulating HMGB1 and Has an Impaired Capacity to Clear Histones in SAP

Proinflammatory mediators are thought to play a crucial role in the pathogenesis of SAP [6, 8, 15, 16, 63, 64]. The amount of cytokines released from the liver represents about 50% of the total cytokine content in the body [64], suggesting that the liver is the major contributor of the circulating cytokines. KCs are the largest number of tissue resident macrophages in the body and a predominant source of circulating inflammatory cytokines in SAP [8], and KCs play a major role in amplifying systemic inflammation during AP [11, 12, 64–68]. Except KCs, emerging evidence is showing that hepatocyte is another important source of circulating HMGB1 in two different experimental SAP models [15, 16]: especially in this triple-hit lethal SAP model, the inflamed liver releases nearly 90% of the hepatic tissue HMGB1 (detected by liver tissue whole cell lysis) [15]. EP is a potent HMGB1 inhibitor that ameliorates SAP via the reduced serum HMGB1 level [17], which is potent in augmenting SIRS in SAP [17, 18]. EP is also found to ameliorate intestinal barrier dysfunction by reducing the ileal mucosal HMGB1 expression [32]. EP not only inhibits LPS-stimulated macrophages to release TNF-*α* and HMGB1 [69], but also inhibits the inflamed liver to release both early (TNF-*α*, IL-6) and late (HMGB1) inflammatory mediators during SAP [15, 16] and resultantly ameliorates the SAP related multiple distant organs injuries (including the liver injury) [6, 15–18]. These evidences indicate that both KCs and the hepatocytes are an important source of circulating HMGB1 in SAP, and the inflamed liver might play a critical role in the translation of pancreatic injury into systemic inflammation and MOD. In addition, the liver is an important organ to rapidly clear extracellular histones [21]; however, SAP can induce severe liver injury and impair its capacity of clearing histones, and this can lead to the increased circulating histones levels, which can significantly contribute multiple organ injury as described above. Therefore, the liver could be a promising therapeutic target to treat SAP.

## 2. Conclusions

Increased circulating HMGB1, histones, and cell-free DNAs levels in SAP patients might play a significant role in contributing to systemic inflammation and multiple organ injury during SAP. HMGB1 is an important factor that might link gut BT and systemic inflammation in SAP. The inflamed liver is an important source of circulating HMGB1 and treating the liver injury is important in SAP.

## Supplementary Material

In SAP, the acinar cell death releases damage associated molecular patterns (DAMPs) such as HMGB1 and histones. HMGB1 contributes to multiple organ injuries and mediates gut BT, BT triggers systemic inflammation that can lead to multiple organ injuries. Histones contribute to multiple organ injuries by damaging endothelial cells. Histones also activate platelets to induce vascular thrombosis, which can lead to MODS.
