# Supplementary material for: HMGB1 and Histones Play a Significant Role in Inducing Systemic Inflammation and Multiple Organ Dysfunctions in Severe Acute Pancreatitis
Source: Int J Inflam. 2017 Feb 21;2017:1817564. doi: 10.1155/2017/1817564 (PMC5339498; doi:10.1155/2017/1817564)
Supplement: Supplementary file 1 — In SAP, the acinar cell death releases damage associated molecular patterns (DAMPs) such as HMGB1 and histones. HMGB1 contributes to multiple organ injuries and mediates gut BT, BT triggers systemic inflammation that can lead to multiple organ injuries. Histones contribute to multiple organ injuries by damaging endothelial cells. Histones also activate platelets to induce vascular thrombosis, which can lead to MODS. [file 1817564.f1.pptx]

## Slide 1
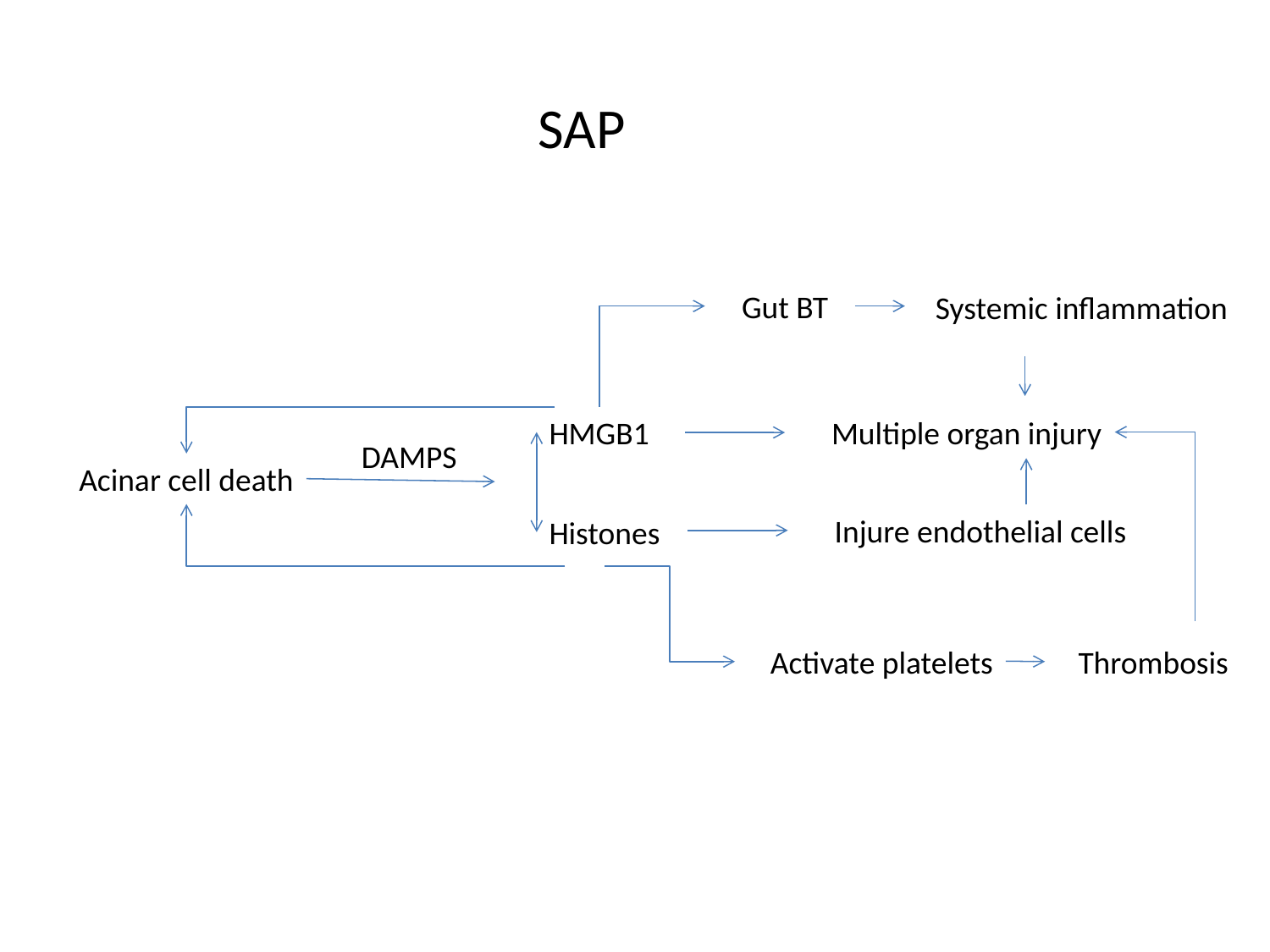

SAP
Gut BT
Systemic inflammation
HMGB1
Multiple organ injury
DAMPS
Acinar cell death
Injure endothelial cells
Histones
Activate platelets
Thrombosis
